# PDIA, an Iminosugar Compound with a Wide Biofilm Inhibitory Spectrum Covering Both Gram-Positive and Gram-Negative Human Bacterial Pathogens

**DOI:** 10.3390/microorganisms10061222

**Published:** 2022-06-15

**Authors:** Łucja Kozień, Estelle Gallienne, Olivier Martin, Sophie Front, Magdalena Strus, Piotr Heczko

**Affiliations:** 1 Department of Bacteriology, Microbial Ecology and Parasitology, Chair of Microbiology, Jagiellonian University Medical College, 31-121 Cracow, Poland; lucja.kozien@uj.edu.pl (Ł.K.); magdalena.strus@uj.edu.pl (M.S.); 2 Institut de Chimie Organique et Analytique (ICOA), UMR 7311, Université d’Orléans & CNRS, rue de Chartres, BP 6759, CEDEX 2, 45067 Orléans, France; estelle.gallienne-boivineau@univ-orleans.fr (E.G.); olivier.martin@univ-orleans.fr (O.M.); sophie.front@univ-orleans.fr (S.F.)

**Keywords:** biofilm, iminosugar PDIA, bacterial human pathogens

## Abstract

Many difficult-to-treat human infections related to catheters and other indwelling devices are caused by bacteria residing in biofilms. One of the key properties of microorganisms residing in a biofilm is decreased susceptibility towards antimicrobial agents. Therefore, many different approaches have been researched to destroy or inhibit biofilm production by bacteria. Different iminosugars (IS) were reported to inhibit biofilm formation in *S. mutans*, *S. aureus,* and *P. aeruginosa*. The aim of this study was to look for a spectrum of the activity in one of these IS. The iminosugar PDIA beta-1-C-propyl-1,4-dideoxy-1,4-imino-L-arabinitol was tested in vitro at the same concentration against 30 different strains of the most important Gram-negative and Gram-positive human pathogens looking for their biofilm production and viability at different time intervals. It appeared that PDIA inhibited biofilm production of *Enterobacter* spp., *P. aeruginosa*, *Enterococcus* spp. and *S. aureus* in 8 h, and *Klebsiella* spp., *Acinetobacter* spp. and *S.epidermidis* in 24 h. PDIA caused no growth inhibition of the tested bacteria at a concentration of 0.9 mM. Our results indicate a broad-spectrum biofilm inhibitory activity of PDIA. which may be the basis for future application studies that will help in control of the associated device and biofilm-related infections caused by a wide spectrum of the causative agents.

## 1. Introduction

Many bacterial infections in humans and animals are caused by bacteria residing in biofilms, which are complex communities of attached organisms embedded in an extracellular matrix. One of the key properties of microorganisms residing in a biofilm is decreased susceptibility towards antimicrobial agents. This is the leading cause for failure of medical implants, resulting in high morbidity and mortality. Biofilms impart enhanced antibiotic resistance and become recalcitrant to host immune responses, leading to persistent and recurrent infections. It makes the clinical treatment for biofilm infections very difficult. Reduced penetration of antibiotic molecules through exopolysaccharides (EPS), mutation of the target site, accumulation of antibiotic degrading enzymes, and enhanced expression of efflux pump genes are the probable causes for antibiotics resistance [1].

This decreased susceptibility, together with conventional mechanisms leading to antimicrobial resistance, makes biofilm-related infections increasingly difficult to treat, and alternative antibiofilm strategies are urgently required. Different strategies to combat biofilm-related infections with the important human pathogens have been proposed and extensively searched. A major focus has been put on various anti-biofilm molecules discovered or tested till date, which may include herbal active compounds, chelating agents, antibacterial peptides, antibiotics, and synthetic chemical compounds [2].

Iminosugars are structural analogues of carbohydrates in which the oxygen atom is replaced by nitrogen. The first natural compound named nojirimycin was discovered 60 years in the 20th century and after that a number of its synthetic derivatives were described. Many natural and synthetic iminosugars are described as inhibitors of glycosidases and glycosyltransferases of both eukaryotic and prokaryotic organisms and, therefore, they show different biological activities [3]. Iminosugars were investigated as potential drugs in a wide range of human diseases like cancer, diabetes, lysosomal storage disorder (i.e., Gaucher disease), or infections caused by HIV and HCV viruses. Concerning bacterial infections, the natural 1-deoxynojirimycin from mulberry leaves was shown to be able to inhibit biofilm formed by *Streptococcus mutans* on teeth as related to dental caries [4]. Following this discovery, iminosugars were considered as effective substances in caries prevention thanks to their possible inhibitory activity against beta-glucan synthesis [5]. We have previously tested several newly synthetized iminosugars for their ability to inhibit biofilm synthesis by *P. aeruginosa* [6]. It appeared that the tested substances inhibited biofilm formation at its early stages but showed no effect on late, already-formed biofilm. The iminosugars did not inhibit bacterial cells propagation at the used concentrations. The mechanism of the biofilm inhibitory effect of these iminosugars remains not fully elucidated, although it is probably related to inhibition of the bacterial enzymes synthetizing bacterial exopolysaccharides. Our preliminary experiments using the same compounds as in the above-cited study [unpublished] suggest that the biofilm-inhibitory activity of iminosugars stretches also on *S. aureus*, a Gram-positive organism of exopolysaccharides structures different from *P. aeruginosa*.

The aim of this study was to check whether the most active of the iminosugars tested in a previous study: PDIA with a marked anti-biofilm activity directed toward *P. aeruginosa,* is also active against other human pathogenic bacteria. The research conducted in this study is basic and is first-phase research on a single candidate substance. A homogeneous biofilm was investigated in which the selected compound acted on a single etiological agent. The results obtained in this study will allow us to move on to a second phase study with a mixed biofilm.

## 2. Materials and Methods

### 2.1. Tested Compound

**Figure 1:**
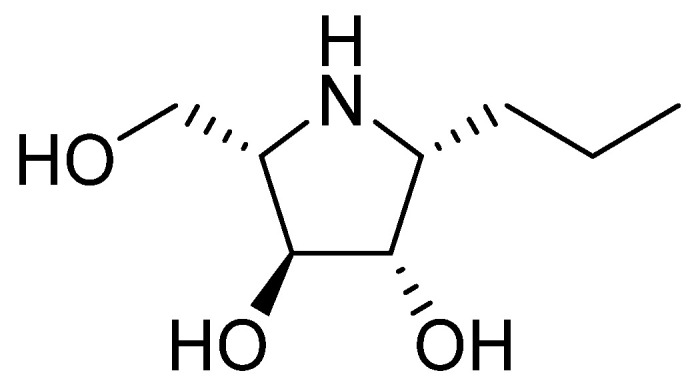
Chemical structure of the iminosugar PDIA.

Investigated iminosugar derivative: PDIA beta-1-C-propyl-1,4-dideoxy-1,4-imino-L-arabinitol was synthesized in the Institute of Organic and Analytical Chemistry at University of Orleans. Its chemical structure is presented in Figure 1, while its synthesis was based on hydrogenation of beta-1-C-allyl-2,3,5-tri-O-benzyl-N-benzyloxycarbonyl-1,4-dideoxy-1,4-imino-L-arabinitol, as described before [6,7].

### 2.2. Bacterial Cultures

Altogether, 29 strains of Gram-positive and Gram-negative bacteria representing 7 different species of the most important human pathogens related to device-associated infections were used. Each species was represented by the collection strain and several clinical isolates. Among these, strains that showed the highest biofilm production capacity were selected and one strain with poor biofilm production from each bacterial group was added as a control. Their taxonomic position was initially tested using standard procedures used in a clinical microbiological laboratory including different API (bioMerieux, Polska) kits/systems and then confirmed with mass spectrometry (MALDI Biotyper, Bruker Scientific LLC, Billerica, MA, USA) according to the manufacturer’s instructions. Actual MBT IVD Library of mass spectra were applied. The pure cultures were kept frozen at −80 °C on glass beads in 2 mL liquid medium Nutrient Broth (Biomaxima, Lublin, Poland) enriched with 15% glycerol (Chempur, Piekary Śląskie, Poland), until used in experiments. All the strains were able to produce biofilm although in different quantities, as tested using the methods described below. Characteristics of the strains are given in Table 1.

Biofilm production intensity was estimated semiquantitatively in grades from + (weak biofilm production), through ++ (medium biofilm production), to +++ (strong biofilm production). The assessment of the intensity of biofilm production was determined separately for each species, due to their differences in the ranges of the absorbance readings of the biofilm measurement.

### 2.3. Growth Conditions and Measurement of Biofilm Formation by Tested Bacteria 

Generally, the method described in our previous paper [6] was used with some modifications related to individual species. Bacterial inoculum was prepared from the frozen pure cultures by incubation of the glass beads coated with bacteria in 10 mL of TSB broth (Beckton Dickinson, Franklin Lakes NJ, USA) and incubation in 37 °C for 24 h. Then, to ensure the purity of the strains, 10 µL aliquots of the cultures were streaked over surfaces of either Columbia Agar (Biomaxima) for Gram-positive bacteria or McConkey Agar (Biomaxima) for Gram-negative bacteria and incubated as before. Three passages were made in the same manner to obtain bacterial populations of high viability. Finally, standardized bacterial suspensions were made by transferring 1 µL of the 24-h broth cultures to 9 mL of saline, vigorous mixing, and adjusting optical density to 0.5 of McFarland scale using a densitometer. This density was adjusted to approximately 1.0 × 10^7^ CFU/mL, as previously controlled with a standard serial dilution method.

Experiments on biofilm production by the tested strains were performed in sterile plastic 96-well plates with an adherent surface (Greiner Bio-One, Kremsmuester, Austria). The bacterial stock suspensions, prepared as described above, in the volume of 20 µL, were placed in each well, then 180 µL of sterile TSB was added, and thus 1 × 10^6^ CFU/well as a final concentration of the bacteria was obtained. The plates were incubated for different times at 37 °C in aerobic conditions after centrifugation for 10 min at 2000 rpm to sediment bacteria on the bottom of each well. Biofilm was measured after staining with Congo red dye (Chempur) according to a modified procedure described by Parai et al. [8]. The plates were removed from incubation at indicated time intervals, and the culture medium was gently pipetted from each well and immediately after 180 µL volume of 0.5 mM Congo red solution was added. The staining was performed for 30 min; the plates were left at room temperature for 20 min and then they were centrifuged for 10 min at 2000 rpm and washed twice with buffered saline to remove unbound dye. The original volume was restored with 180 µL of saline and absorbance was measured using spectrophotometer (Tecan Sunrise, Mannedorf, Switzerland) at 492 nm wavelength. Measurements for all strains were performed in triplicates and mean values ± SD were calculated.

### 2.4. Measurement of the Iminosugar Effect on Early and Mature Biofilm and on Bacterial Cells

The effect of PDIA on the production of biofilm by bacterial cells was measured as follows. At first, by 96-well plate were filled with 20 µL of freshly prepared suspensions of the tested strains containing 1 × 10^7^ CFU/mL and 180 µL of TSB broth (Becton Dickinson). Eight wells were inoculated with a single strain tested: six to check biofilm formation in three time intervals under the influence of the PDIA and in control wells, and next two for measuring the number of viable bacteria. The plates were incubated at 37 °C as described above for 4 h. Then, 180 µL of the bacterial culture was pipetted out of each well and 180 µL of the PDIA at a final concentration of 0.9 mM was added. Fresh TSB medium w/o test substance was added to the control wells. The plates were gently rotated to distribute the iminosugar and incubated for different times at 37 °C in aerobic conditions. Parallel wells were removed in the following time intervals: 4, 8, and 24 h. The method of measuring biofilm using Congo red was applied as described above. At the same time intervals, bacterial cultures in each well of the second row were as mixed by multiple pipetting and transferred to sterile tubes. The decimal dilutions of the bacterial suspensions were made in tubes with 0.9 mL saline. The 100 µL volumes of the dilutions were plated on Columbia Agar (Biomaxima) for Gram-positive bacteria or McConkey Agar (Biomaxima) for Gram-negative bacteria, spread over the surface and incubated at 37 °C for 24 h. Numbers of colonies grown on plates were counted and total numbers of the viable bacteria were calculated as colony forming units (CFU) per ml.

Effect of PDIA on mature biofilm formation was measured the same way as for early biofilm, but before adding PDIA at the concentration of 0.9 mM, the wells with bacterial cultures were incubated for 24 h. The biofilm formation and the number of viable bacteria were checked in the following time intervals: 24, 24 + 8, and 48 h after the addition of the iminosugar to 24-h bacterial cultures. Biofilm quantity and numbers of viable bacterial cells embedded in mature biofilm were checked as described above.

### 2.5. Statistical Analysis

STATISTICA, version 10 software was used to make statistical analyses based on Student’s t-test to compare values for bacterial strains within the genus or species and then between mean values for all tested strains within a taxon versus control. Significant differences were assumed when *p* was <0.05 and highly significant when *p* was <0.01.

## 3. Results

All tested strains responded to PDIA iminosugar with inhibition of the biofilm production. Differences among strains of the same genus/species were calculated to observe possible individual variations in their susceptibility to biofilm-inhibitory properties of the tested iminosugar. These differences appeared to be non-significant. Therefore, further comparisons were made with groups of bacteria either of the same genera—when a few strains from different species of the genus were tested, or with the entire bacterial species—when more strains represented them.

**Figure 2:**
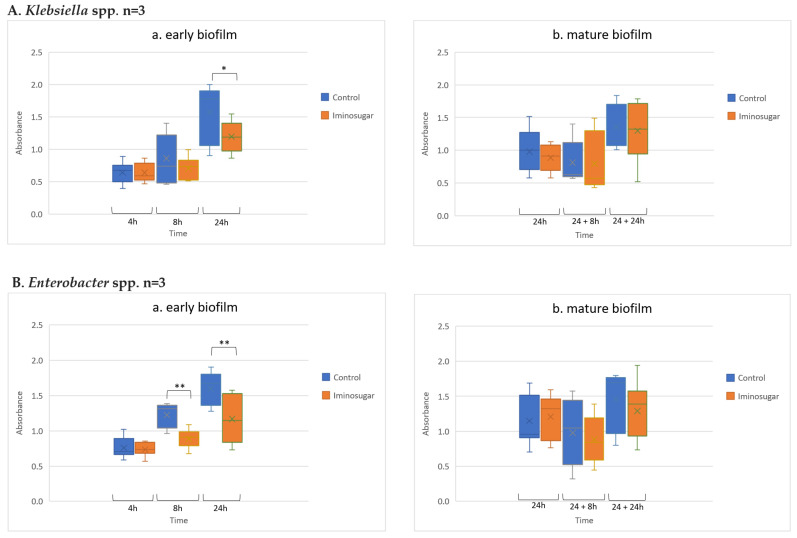
Effect of iminosugar PDIA on biofilm production by selected Gram-negative *Enterobacteriaceae*. (**A**), *Klebsiella* spp., (**B**), and *Enterobacter* spp. in (**a**) early biofilm and (**b**) mature biofilm. n refers to the number of strains. The figure represents biofilm absorbance readings for all strains belonging to a given species at a given time (measurements for each strain were performed in triplicates). Biofilm production expressed as absorbance (λ = 492 nm) after Congo red staining at different time points. * *p* < 0.05 and ** *p* < 0.01.

**Figure 3:**
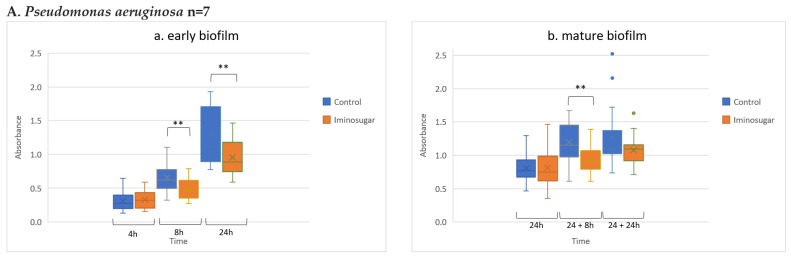

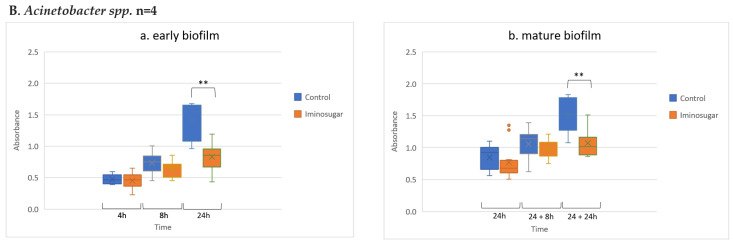
Effect of iminosugar PDIA on biofilm production by selected Gram-negative non-fermentive rods. (**A**) *Pseudomonas aeruginosa* and (**B**) *Acinetobacter* spp. in (**a**) early biofilm and (**b**) mature biofilm. n refers to the number of strains. The figure represents biofilm absorbance readings for all strains belonging to a given species at a given time (measurements for each strain were performed in triplicates). Biofilm production expressed as absorbance (λ = 492 nm) after Congo red staining at different time points. ** *p* < 0.01.

**Figure 4:**
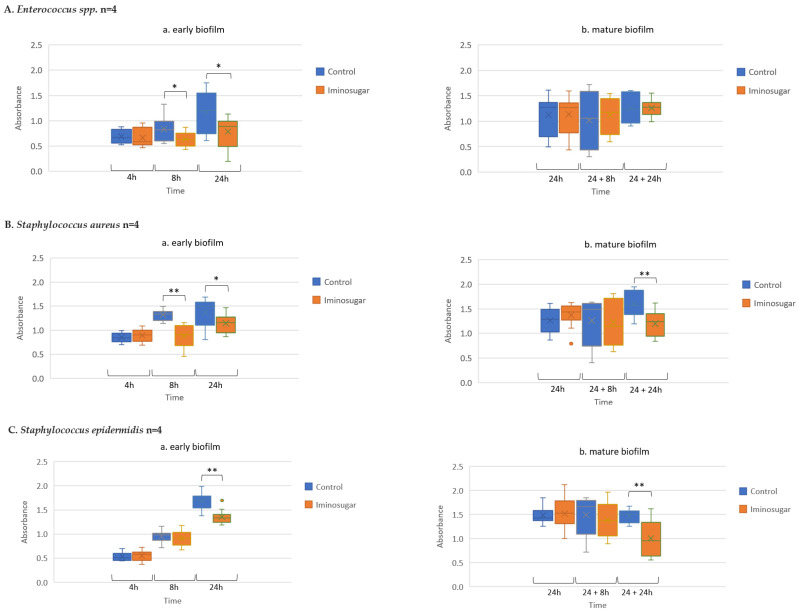
Effect of iminosugar PDIA on biofilm production by selected Gram-positive cocci. (**A**) for *Enterococcus* spp. (**B**), *Staphylococcus aureus*, and (**C**) *Staphyloccous epidermidis* in (**a**) early biofilm and (**b**) mature biofilm. n refers to the number of strains. The figure represents biofilm absorbance readings for all strains belonging to a given species at a given time (measurements for each strain were performed in triplicates). Biofilm production expressed as absorbance (λ = 492 nm) after Congo red staining at different time points. * *p* < 0.05 and ** *p* < 0.01.

Results are given in Figure 2 for Gram-negative rods, Figure 3 for Gram-negative non-fermenting rods, and Figure 4 for Gram-positive cocci. Significant differences were observed for inhibition of the early biofilm production in comparison to control by all tested bacterial genera/species. The inhibitory effect of PDIA was visible in already 8 h of incubation of Gram-negative bacteria from genus *Enterobacter* and *Pseudomonas aeruginosa* species, and from genus *Enterococcus* and *Staphylococcus aureus* species. Inhibition of the early biofilm formation of the remaining bacteria: *Klebsiella* spp., *Acinetobacter* spp., and *S.epidermidis* was prolonged for 24 h. The effect was also noted for late or mature biofilm production by some bacteria taxons: *P.aeruginosa* in 24 + 8 h, and for *Acinetobacter* spp., *S.aureus,* and *S.epidermidis* at 48 h. 

**Figure 5:**
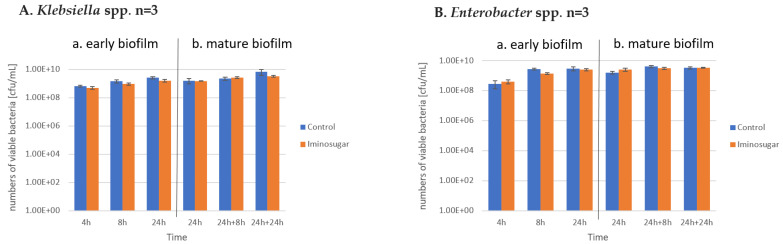
Effect of the tested iminosugar PDIA on numbers of viable population (n. refers to the number of strains) of selected Gram-negative *Enterobacteriaceae* (**A**) for *Klebsiella* spp. and (**B**) *Enterobacter* spp. in early (**a**) and mature (**b**) biofilm phase formation. Results depict logs of CFU/mL and measured at different time intervals. CFU, colony-forming units.

**Figure 6:**
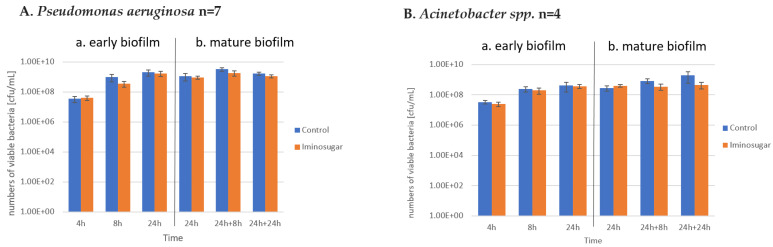
Effect of the tested iminosugar PDIA on numbers of viable population (n refers to the number of strains) of selected Gram-negative non-fermentive rods for (**A**) *Pseudomonas aeruginosa* and (**B**) *Acinetobacter* spp. in early (**a**) and mature (**b**) biofilm phase formation. Results depict logs of CFU/mL and measured at different time intervals. CFU, colony-forming units.

**Figure 7:**
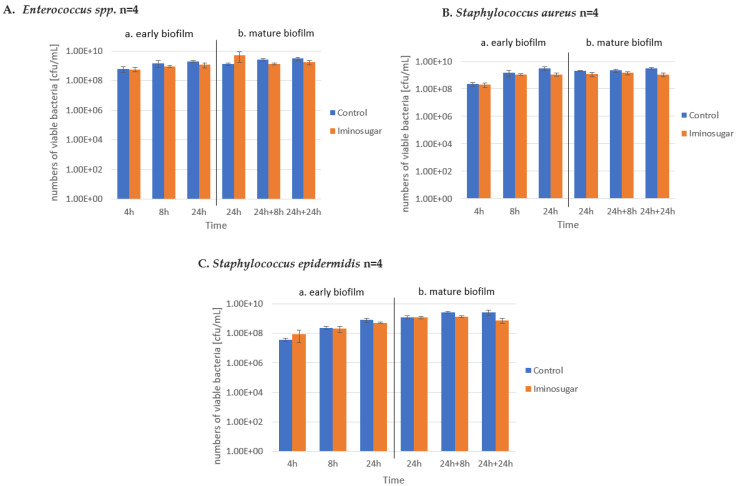
Effect of the tested iminosugar PDIA on numbers of viable population (n refers to the number of strains) of selected Gram-positive cocci for (**A**). *Enterococcus* spp. and (**B**) *Staphylococcus aureus*, and (**C**) *Staphylococcus epidermidis* in early (**a**) and mature (**b**) biofilm phase formation. Results depict logs of CFU/mL and measured at different time intervals. CFU, colony-forming units.

PDIA at the used concentration had no effect on the number of viable bacteria during the observation period, as shown in Figure 5, Figure 6 and Figure 7. It was observed that the number of bacteria over time increased after 24 h and then remained at a similar level with no clear differences and statistical significance.

## 4. Discussion

Undoubtedly, the most important information derived from this study is that a single iminosugar compound PDIA showed a very wide spectrum of its activity as inhibitor of the biofilm production by 7 different species of both Gram-positive and Gram-negative human bacterial pathogens, which cause the vast majority of hospital infections [9,10]. What is even more important is that all these bacteria are related to so-called device-related infections in which the mechanisms of biofilm are one of the pathogenicity factors [11,12].

In our previous paper, we demonstrated that several tested iminosugars inhibited early biofilm formation by *P. aeruginosa* without any effect on already-formed, mature biofilm [6]. The explanation can be found in methodological differences between these two studies; the development time of early and mature biofilm is different, moreover, the synthesis conditions were slightly modified from the previous ones. The reason for this is to eliminate the measurement error resulting from the effect of sticky mucus formation in cultures incubated for more than 48 h. This mucus disturbed the biofilm structure during pipetting, and thus disturbed the correct absorbance reading. In addition, all procedures on early biofilm were started 4 h after culture set up to allow the bacteria to better adhere to the well surface.

The results of our study confirm that the effect of the tested iminosugar on bacterial biofilm is species- but not strain-dependent. Additionally, critical points were found among the seven bacterial groups where the early biofilm was inhibited, indicating its wide spectrum of action, including both Gram-positive and Gram-negative bacteria. Although inhibition of biofilm production does not occur consistently at all stages of fresh and mature biofilm formation, the points at which it occurred may be the subject of further and more detailed studies leading to complete eradication of the bacterial biofilm. Thanks to the practical lack of the bacteriostatic properties of the tested iminosugar, we know that the basis of its biofilm-inhibitory action is as shown in other studies, blocking the enzymes involved in exopolysaccharides synthesis [13].

The conducted experiments are first-phase studies. Therefore, a single-species biofilm was only studied. Up to now, all reports on biofilm inhibitory activity of the iminosugars were limited to single bacterial species like as *S. aureus* [14], *S. epidermidis* [15], *S. mutans* [4], or *P. aeruginosa* in our case [6].

Unfortunately, the mechanism of the PDIA biofilm formation inhibition remains unknown, since this group of iminosugars has never been tested against putative bacterial enzymes involved in the synthesis of the biofilm exopolysaccharide matrix, which is the most probable target. Conforti and Marra [13], in their recently published review on iminosugars as glycosyltransferase inhibitors, did not mention PDIA, although they included similar pyrrolidine derivatives. It is likely that also this compound can act as a glycosyltransferase inhibitor, but data on the inhibition of the enzymes involved in the biosynthesis of exopolysaccharides by such compounds are still lacking.

The tested substance PDIA had no effect on viability and propagation of the bacterial cells at least at the used concentration of 0.9 µM (158 µg/mL). De Gregorio et al. [14] reported recently that a modified natural iminosugar nojirimycin namely N-Nonyloxypentyl-1-Deoxynojirimycin inhibited both the growth and biofilm formation of collection and clinical strains of *S. aureus* in a dose-dependent manner, with an MIC value of 128 µg/mL. It should be noted that the structure of the compound tested by De Gregorio is considerably different from PDIA. On the other hand, very high MIC/MBC values of the most effective iminosugar tested by De Gregorio make a therapeutic use of this compound rather improbable. As demonstrated in our previous study, PDIA and related compounds were, at the used concentration, not cytotoxic against human cells in vitro, which may be to their advantage in the case of their medical application.

The concentration of PDIA iminosugar was selected on the basis of previously mentioned unpublished PhD studies and our previous publication [6]. Thus, the selection of the optimal iminosugar test concentration was based on experimental and literature data. Also, as written in the introduction, the same concentration was effective against *S. aureus* biofilm in our pilot study. Thus, the same concentration of the compound active against *Staphylococcus* and *Pseudomonas aeruginosa* strains was selected to test other species. The results obtained for *Pseudomonas aeruginosa* strains are presented in the cited publication [6]. Further studies on defining the exact minimal biofilm inhibitory values for different species are obviously planned.

As mentioned before, biofilm-mediated infections and especially those related to indwelling devices are difficult to control due to their complexity and antibiotic resistance. Thus, early prevention of their surface colonization to restrict biofilm development, as the first step in the formation of biofilms, seems to be of particular importance. Therefore, it is possible to speculate that a probable application of the PDIA compound in preventing biofilm formation by, for example, coating surfaces of the medical devices, may well help in the control of the associated device and biofilm-related infections caused by a wide spectrum of the causative agents [16].

## 5. Conclusions

Many different biofilm inhibitors have been investigated in order to prevent biofilm formation and eliminate persistent biofilms. In spite of these attempts, the research focused on identifying compounds able to target and inhibit this biofilm mode of bacterial growth, which is explicitly still inadequate. Iminosugars, and especially PDIA, should be considered as a group of bacterial biofilm inhibitors that are useful test substances for in future in-vivo studies on animal models of the device-associated infections. It is possible to speculate that a probable application of the PDIA compound in preventing biofilm formation can be achieved by, for example, coating surfaces of medical devices.

## Figures and Tables

**Table 1:** Bacterial strains used.

Species	Collection/Accession Number	Biofilm Production	Origin	Type of Strain
*Acinetobacter baumannii*	ATCC 19606	+++	ATCC ^1^	Type strain
*Acinetobacter baumannii*	746	+	CNS infection	Clinical isolate
*Acinetobacter baumannii*	7	+++	Pancreatic infection	Clinical isolate
*Acinetobacter baumannii*	415	+++	Sepsis/blood	Clinical isolate
*Enterobacter cloacae*	28	++	Bronchial lavage	Clinical isolate
*Enterobacter cloacae*	223	++	Sepsis/blood	Clinical isolate
*Enterobacter cloacae*	74	+++	Sepsis/blood	Clinical isolate
*Enterococcus faecalis*	PCM 2605	+++	PCM ^2^	Type strain
*Enterococcus gallinarum*	19B	+	Crohn disease	Clinical isolate
*Enterococcus faecalis*	31B	+++	Crohn disease	Clinical isolate
*Enterococcus faecalis*	48A	+++	Ulcerative colitis	Clinical isolate
*Klebsiella pneumoniae*	20	+	Tracheal aspirate	Clinical isolate
*Klebsiella pneumoniae*	230	+++	Tracheal aspirate	Clinical isolate
*Klebsiella pneumoniae*	236	+++	UTI ^3^	Clinical isolate
*Pseudomonas aeruginosa*	PCM 2562	+++	PCM ^2^	Type strain
*Pseudomonas aeruginosa*	2	+	Cystic fibrosis	Clinical isolate
*Pseudomonas aeruginosa*	5	+++	Diabetic foot infection	Clinical isolate
*Pseudomonas aeruginosa*	50	+++	Diabetic foot infection	Clinical isolate
*Pseudomonas aeruginosa*	95B	+++	Cystic fibrosis	Clinical isolate
*Pseudomonas aeruginosa*	91	+++	Cystic fibrosis	Clinical isolate
*Pseudomonas aeruginosa*	28	+++	Cystic fibrosis	Clinical isolate
*Staphylococcus aureus*	PCM 2602	++	PCM ^2^	Type strain
*Staphylococcus aureus*	46	++	Otitis media	Clinical isolate
*Staphylococcus aureus*	48	+++	Otitis media	Clinical isolate
*Staphylococcus aureus*	72	+++	Otitis media	Clinical isolate
*Staphylococcus epidermidis*	PCM 2118	+++	PCM ^2^	Type strain
*Staphylococcus epidermidis*	284	+	Blood; sepsis	Clinical isolate
*Staphylococcus epidermidis*	192	+++	Blood; sepsis	Clinical isolate
*Staphylococcus epidermidis*	263	+++	Blood; sepsis	Clinical isolate

^1^ ATCC: American Type Culture Collection; ^2^ PCM: Polish Collection of Microorganisms; ^3^ UTI, urinary tract infection.

## Data Availability

Data available on request from the corresponding author.
